# Adipose endothelial cells mastering adipose tissues metabolic fate

**DOI:** 10.1080/21623945.2022.2028372

**Published:** 2022-01-22

**Authors:** Zhe-Zhen Liao, Li Ran, Xiao-Yan Qi, Ya-Di Wang, Yuan-Yuan Wang, Jing Yang, Jiang-Hua Liu, Xin-Hua Xiao

**Affiliations:** The First Affiliated Hospital of University of South China, Department of Metabolism and Endocrinology, Hengyang Medical School, University of South China, Hengyang, Hunan, China

**Keywords:** Obesity, adipose endothelial cells, communication, adipocytes

## Abstract

Dynamic communication within adipose tissue depends on highly vascularized structural characteristics to maintain systemic metabolic homoeostasis. Recently, it has been noted that adipose endothelial cells (AdECs) act as essential bridges for biological information transmission between adipose-resident cells. Hence, paracrine regulators that mediate crosstalk between AdECs and adipose stromal cells were summarized. We also highlight the importance of AdECs to maintain adipocytes metabolic homoeostasis by regulating insulin sensitivity, lipid turnover and plasticity. The differential regulation of AdECs in adipose plasticity often depends on vascular density and metabolic states. Although choosing pro-angiogenic or anti-angiogenic therapies for obesity is still a matter of debate in clinical settings, the growing numbers of drugs have been confirmed to play an anti-obesity effect by affecting vascularization. Pharmacologic angiogenesis intervention has great potential as therapeutic strategies for obesity.

## Graphical abstract

**Figure:**
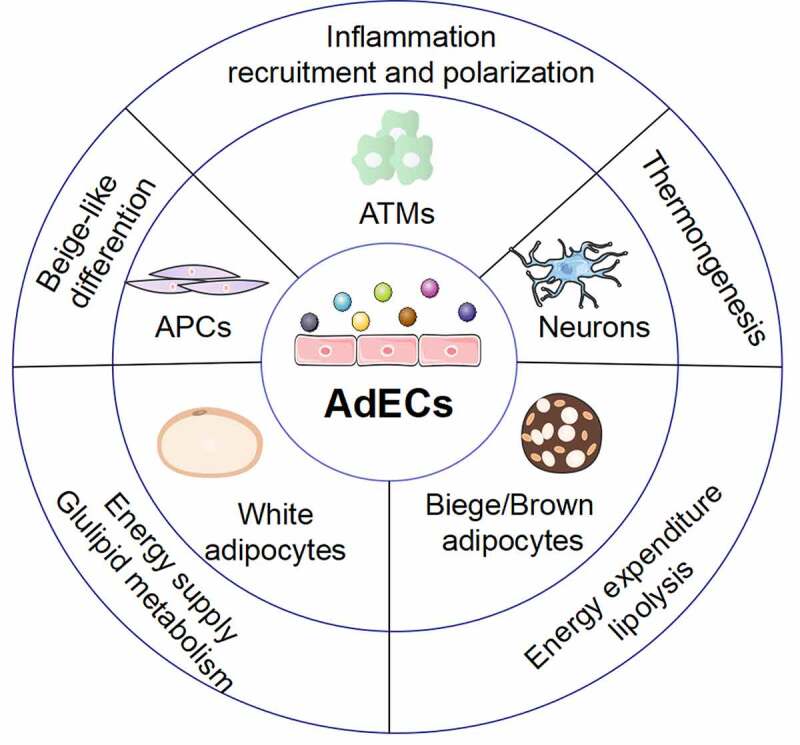

## 1. Introduction

Obesity severely affects the quality of life and threatens the health of patients because of the accelerated growth rate and serious complications [1]. The traditional opinion of the pathogenesis of obesity is centred on the response of adipocytes to insulin resistance (IR) and lipotoxicity [2]. Immune system dysfunction and vascular impairment are considered secondary events [2]. However, the importance of vascular pathogenesis as an initial trigger for the development of obesity and its comorbidities has been gradually recognized [3]. Adipose endothelial cells (AdECs) form a network of capillaries with a total length of nearly 1 m in each cubic millimetre of adipose tissue (AT) [4]. Each fat cell is in direct contact with the capillary network in a circular manner. AdECs are not only a physical barrier between blood and the other cells of AT but also directly regulate the effective transport of oxygen, nutrients, hormones, etc. in AT, and thereby have a lasting impact on the balance of AT metabolism [4–6]. Unexpected findings have transformed our understanding of the role of AdECs in the development of obesity prevention strategies that target AdECs [7,8].

Herein, we summarize and discuss the dramatically underexplored evidence in favour of the causal role of AdECs in systemic metabolic control. This review provides an overview of the molecular mechanisms of communication between AdECs-adipocyte and AdECs-stromal cells in the control of AT metabolic homoeostasis.

## 2. The metabolic characteristics of different types of AdECs

Endothelial cells in ATs can be divided into arterial endothelial cells (AECs), venous endothelial cells (VECs), capillary endothelial cells (CECs) and lymphatic endothelial cells (LECs) based on anatomy locations [9]. AECs are directly involved in maintaining the physiological functions of AT through local blood flow regulation and nutrient supply. VECs are the primary site of permeability and recruitment of chemokines during obesity-induced inflammation [9]. However, this insufficiency in knowledge of regulations of AECs and VECs in AT metabolism should be subject for further examination. The lower capillary density in the white adipose tissues (WAT) of patients with obesity could impair the lipids-storing capacity of WAT, and then lead to ectopic fat accumulation [10]. The impaired thermogenesis of brown adipose tissues (BAT) was also correlated with capillary rarefaction in obese mice 11. The results of single-cell sequencing from visceral AT in obese subjects recently provided new clues to the types of AdECs, which indicates that 78% of vascular stromal cells from the visceral AT expressed genes *LYVE1* [12–14]. *LYVE1* as a classic marker of LECs is enriched in AdECs, implicating that AdECs may present LECs-like metabolic and structural features. LECs have been reported to control lipid transport and mediate AT inflammation via regulation of intestine-adipose axis [15–19]. This observation keeps line with the recent literature showing the crosstalk between LECs and AT function [20]. Although important advances have been made about types of AdECs in WAT, very little progress has been achieved for precise classification of AdECs in other fat pads.

## 3. AdECs signalling networks within adipose stromal cells

**Figure 1.**
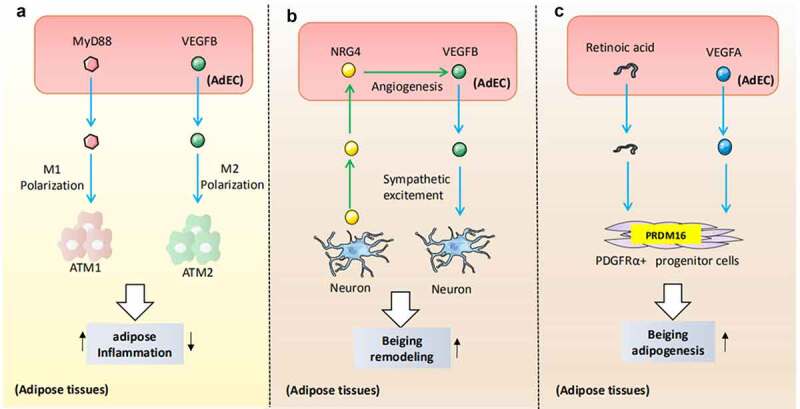
The crosstalk of AdECs and adipose stromal cells. A) VEGFB or MyD88 released from AdECs determines the polarization direction of adipose tissue macrophages. B) AdECs engage in unique interactions with sympathetic neurons. VEGF-B release from AdECs binds to VEGF-R in sympathetic neurons to increase sympathetic innervation, which further promotes beige adipocyte formation. In turn, the sympathetic neuron-derived NRG4 induces AdECs to secrete VEGF-B, creating a positive feedback loop. C) Retinoic acid and VEGF-A signalling mediated-high vascularization supply sufficient nutrients to adipose progenitor cells and subsequently accelerates PDGFRα+ progenitor cells to differentiate them into beige adipocytes by binding to the promoter of PRDM16.

AdECs can secrete signalling molecules to target neuron cells, immune cells, and progenitors, altering their metabolic pathways and reshaping the AT microenvironment. In this study, we will comprehensively summarize the current studies on molecular signalling linking crosstalk between AdECs-immune cells, AdECs-neuron cells, and AdECs-progenitor cells (Figure 1).

### 3.1 AdECs- immune cells communication

A variety of AdEC-derived factors may target adipose-resident immune cells to regulate adipose metabolism and inflammatory responses [21]. Adipose tissue macrophages (ATMs) are one of the most abundant immune cell types present in visceral AT and can be categorized as M1-like (pro-inflammatory) and M2-like macrophages (anti-inflammatory) [22]. Obesity drives macrophages to polarize into an M1-like phenotype; strategies to increase the ratio of the M2-dominant population can improve adipose and whole-body metabolism [23]. Interestingly, modulation of angiogenesis can also cause a change in ATM phenotype. Vascular endothelial growth factor B (VEGF-B), the best-known angiogenic factor, binds to VEGFR1 on the surface of macrophages, causing M2-macrophage polarization [24]. Although the mechanism by which vascularization is prone to polarize ATMs towards M2-like macrophages remains to be explored, local vasculature administration could be a potential clinical route to improve AT inflammation. On the other hand, obesity-damaged AdECs could directly activate endothelial TLR-MyD88 signalling to drive M1 macrophages accumulation through production of GM-CSF, which is well known for promoting monocyte differentiation towards M1 pro-inflammatory macrophages [25]. Moreover, ATMs could regulate the formation of AdECs by producing po-angiogenic factors, such as the platelet-derived growth factor-B (PDGF-B) [26]. These findings suggest that AdECs harbour immunosuppressive feedback mechanisms that are triggered as a result of macrophage activation. Disturbances in AdEC–macrophage interactions drive the occurrence of obesity. We look forward to more research involving the interaction of AdECs with other immune cells in obesity because the current investigations have been confined to the communication between AdECs and ATMs.

### 3.2 AdECs- neural cells communication

The close connection between AdECs and sympathetic stimuli might be essential for AT remodelling during overfeeding or cold-induced thermogenesis [27]. AdECs-derived VEGF-B or VEGF-A can increase sympathetic control by binding to the VEGFR presented on the surface of sympathetic neurons [28,29]. Subsequently, excited sympathetic neurons might secrete noradrenaline or neuregulin 4 (NRG4) to activate β-adrenergic receptors in the AdECs to induce VEGF production, which could elicit beiging in white adipocytes, along with by increased microvascular density [30]. Meanwhile, the abnormal secretion of sympathetic neurotransmitters could initiate the lipolysis of adipocytes and the release of inflammatory adipokines, which reshape the function of AdECs [31]. Apart from the interaction between the peripheral sympathetic nerve cell system and the AdECs, the regulatory role of the peripheral vagus nerve on AdECs still requires further exploration.

### 3.3 AdECs- adipocyte progenitor communication

AT is a cellular heterogeneous endocrine organ. In addition to adipocytes, adipose progenitor cells (APCs), which can create mature adipocytes, actively participate in metabolic homoeostasis [31]. APCs reside in a state of relative quiescence during adulthood [32]. The mechanisms responsible for establishing an activated state of APCs are closely related to vascular network formation. High vascularization supplies sufficient nutrients to APCs, and subsequently accelerates the mobilization of APCs that differentiate to produce new adipocytes [33]. Accordingly, the activation of peroxisome proliferator-activated receptor γ (PPARγ)-VEGF signalling promotes the recruitment of APCs for adipogenesis and endothelial cell proliferation, highlighting the importance of neovascularization in the adipogenic differentiation of APCs [34]. Importantly, in visceral fat, a subpopulation of APCs, known as beige-like adipogenic progenitors (PDGFRα^+^), could be triggered and differentiated into beige adipocytes by retinoic acid (RA), a metabolite of vitamin A [35]. The pro-beiging effect of RA on PDGFRα^+^ progenitor cells requires the upregulation of the VEGF signalling pathway to bind to the promoter of PRDM16, which is a transcription factor that determines the fate of brown and beige fat [35]. Hence, exploring the key factors for beige-like differentiation and to unlock the commitment potential of APCs could be a promising strategy to combat obesity.

## 4. AdECs: the gatekeeper of adipocyte metabolism

**Figure 2.**
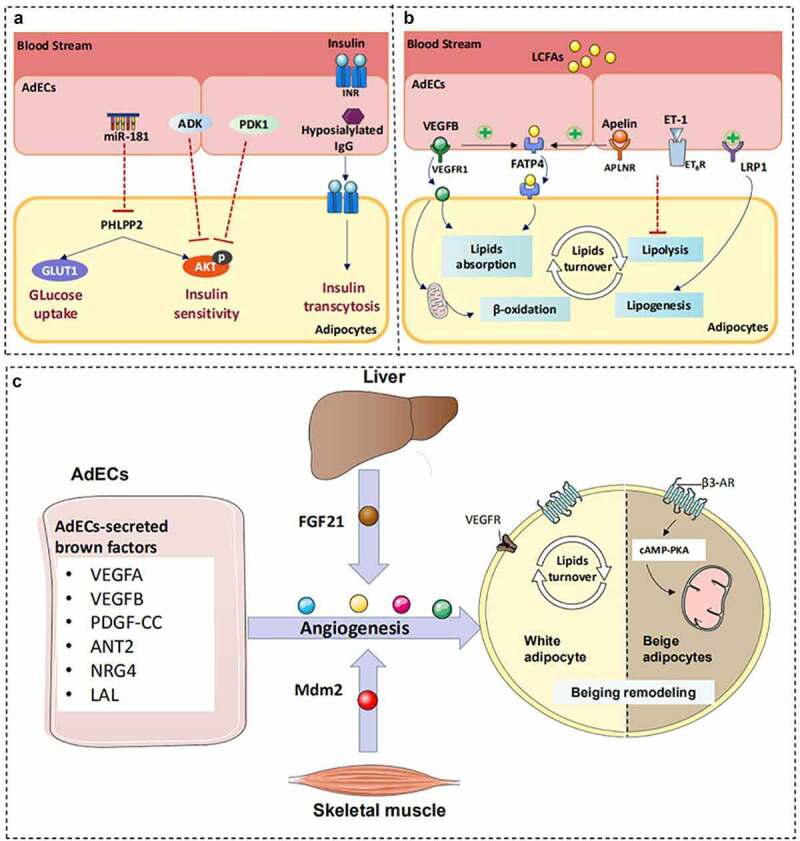
Proposed paracrine mechanism whereby AdECs modulate adipose metabolism. A) AdECs-derived factors, including hyposialylated IgG, ADK, and PDK1, can modulate insulin sensitivity by regulating insulin exocytosis, insulin signal transduction, and glucose uptake. B) VEGF-B, LRP1, apelin, and ET-1 are essential modulators for adipose lipid metabolism and lipid turnover. These factors released by AdECs regulate the expression of key elements required for lipolysis, lipogenesis, and β-oxidation in adipocytes, which further contributes to unhealthy adipose expansion. C) The angiogenic switch triggers beige remodelling based on the secretion of AdECs derived from brown mediators. The angiogenesis of AdECs causes WAT browning and energy expenditure via activation of VEGFR and activation of β3-AR. Liver and muscle release FGF21 and MDM2 and target AdECs for beiging initiation.

Abnormal communication originating from AdECs or adipocytes leads to obesity and associated complications [36]. Pellegrinelli et al. were the first to highlight that AdEC dysfunction negatively disrupts adipocyte metabolic balance by lowering insulin sensitivity, triggering metabolic stress, and promoting the release of pro-inflammatory cytokines in patients with obesity [37]. Hence, we comprehensively summarize the multiple mechanisms by which AdECs modulate adipocyte IR, lipid overload, and metabolic remodelling (Figure 2).

### 4.1 AdECs: active players of adipocyte insulin sensitivity

In recent years, our understanding of the signalling molecules controlling insulin and glucose transport across the AdECs has unremitting increased. Examples of AdECs-derived protective factors include miR-181 and IgG receptor FcgRIIB, which serve as powerful regulators of insulin sensitivity and are implicated in the maintenance of metabolic homoeostasis in AT [38–41]. MiR-181 produced by AdECs enhance insulin signalling transduction through AKT phosphorylation and by inducing the expression of glucose transporter proteins in adipocytes [38]. Activation of the immunoglobulin (Ig) G receptor FcgRIIB and in the AdECs by hyposialylated IgG impedes endothelial insulin transcytosis into adipocytes, revealing the exact mechanism of insulin transport between AdECs and adipocytes [40]. AdECs also release detrimental factors PDK and ADK to directly impair adipocyte insulin sensitivity and glucose levels by inhibiting AKT activity [39,41]. Collectively, these findings suggest that the dysregulation of AdECs directly impairs insulin sensitivity and disrupts glucose homoeostasis within the AT.

### 4.2 AdECs: active players of adipocyte lipids turnover

The functional relevance of adipocyte-AdEC circuitry for adipose lipid turnover was also demonstrated in studies designed to explore the function of AdECs in the maintenance of adipocyte lipid homoeostasis. AdECs have a tight cross-talk with adipocytes for fatty acid (FA) turnover as they secrete VEGF-B and apelin, which promote endothelial FA absorption by modulating FATP4 or CD36 and transport lipids into adipocytes [42–44]. This lipid transport is mediated by VEGFR1 and the apelin receptor APLNR, which are expressed by the AdECs, and have critical roles in coordinating AdECs-mediated FA uptake and the energy demand of the surrounding adipocytes [42,43]. Moreover, VEGF-B is co-expressed with mitochondrial proteins to coordinate FA β-oxidation in adipocytes and reduce the lipid accumulation in adipocytes [43]. Recently, a low-density lipoprotein receptor-related protein 1 (LRP1) was reported to induce lipogenesis by activating PPARγ signalling in white adipocytes [44,45]. The molecular mechanism of AdECs for controlling lipid catabolism was also demonstrated to involve endothelin-1, which, as an angiogenesis inhibitor, could suppress hormone-sensitive lipase-mediated lipolysis of adipocytes [46,47]. In summary, AdECs are highly specialized endothelial cells that are adapted to regulate lipid transport and lipid metabolism in adipocytes, and are implicated in the pathogenesis of obesity.

### 4.3 Angiogenesis of AdECs linking adipocytes remodelling: friends or foes?

AdECs are the most important cells for AT vasculature involved in self-renewal and construction of the lumen and basement membrane [48]. Most studies have reported that AdEC-dependent angiogenesis differentially regulates the metabolism of white and brown fat.

#### Angiogenesis of AdECs favours activation of brown/beige adipocytes

Beige-like or brown-like AT remodelling is an energy-consuming process that relies on the growth of microvessels to match the energy demands [49]. The angiogenesis of AdECs could induce the activation of beige and brown adipocytes and accelerate energy consumption [50–53]. Furthermore, the delivery of pro-angiogenic factors to metabolically active brown or beige adipocytes may accelerate fat burning and improve insulin sensitivity [49–51]. Pro-angiogenic factors released by AdECs could trigger a browning programmes via activation of VEGFR and the β3-AR [54,55]. Such factors play promoting roles in browning and angiogenesis and are termed brown-angiogenic factors.

In addition to paracrine factors derived from AdECs that can regulate vascularization and the browning process, the fibroblast growth factor-21 (FGF21) and murine double minute 2 (Mdm-2) derived from the liver and skeletal muscles can change the phenotype of adipocytes by regulating AdEC-dependent angiogenesis [56,57]. Recently, an outstanding study revealed a novel molecular mechanism for the AdEC-governed fat cell thermogenic fate [58]. The endocytosis of triglyceride-rich lipoprotein (TRL) particles by AdECs through the lysosomal acid lipase (LAL) pathway contributes to the browning of white adipocytes [58]. However, the paracrine molecules secreted by AdECs during intracellular TRL processing remain to be elucidated. Further studies are essential to identify the biological functions of these potential molecular mediators and to explore the proposed AdEC-adipocyte communication.

#### Angiogenesis of AdECs in metabolism of white adipocytes: a double-edged sword

Obesity causes excess energy to be deposited in the white adipocytes, and its progression leads to a high demand for oxygen and nutrients [59]. Hence, strategies to block oxygen and nutrient supply by preventing white adipocyte angiogenesis could combat obesity [59,60]. Based on the theoretical notions above, some angiogenesis inhibitors, such as endostatin and *AGO1*, have been found to resist obesity [60–66], whereas proangiogenic factors such as ANT-2 exert anti-obesity effect [67].

Certain rodent literature held the opposite views that promoting angiogenesis can effectively improve local hypoxia, fibrosis and inflammation induced by the rapid expansion of fat cells. Conversely, targeted delivery of pro-angiogenic factors including VEGFs, PDGF-B and PDGF-CC into AT enhances microvessel growth and can effectively reverse adiposity, macrophage infiltration, and oxidative stress [68–70]. Hence, the AT phenotype may depend on the metabolic state of the white adipocytes. During the metabolic quiescent state, excessive angiogenesis leads to an obese phenotype, while during the metabolically active phase or metabolic stress, excessive angiogenesis leads to a lean phenotype.

#### Pharmaceutical angiogenesis intervention for combating obesity

In the previous chapter, we have introduced the pros and cons of angiogenesis on fat metabolism in animal experiments. Limited AT vascularization and blood flow were shown to be correlated with hypoxia and insulin resistance, whereas hyperactived angiogenesis may result in unhealthy expansion of WAT [71]. Thus, angiogenesis of AT is a potential target for metabolic diseases. Currently, it remains obscure whether angiogenesis stimulation or inhibition serves as a treatment for obesity. Hence, we summarize clinical evidence describing angiogenesis activity in obesity, which provides a rationale for angiogenesis option (Table 1) [72–74]. In a randomized controlled study with a small sample size, postmenopausal women with obesity had higher serum levels of angiogenic factors, such as VEGF, plasminogen activator inhibitor, and pigment epithelium-derived factor [71]. Other studies found that weight loss programmes, including exercise or diet intervention, lead to decreased or increased angiogenesis activity in the circulation or AT [72–76]. Furthermore, we have also summarized the new mechanisms of other drugs to improve obesity-associated metabolic disorders from the perspective of angiogenesis, although most of the research on these drugs is still limited to animals.

**Table 1.** Clinical correlation between angiogenesis and obesity

Drug or diet or exercise therapy	Change of angiogenesis markers	Conclusion	Disease (sample size)
Exercise /Diet-induced weight loss			
Diet intervention or Exercise intervention^[71]^	Circulating VEGF ↓ Circulating PEDF ↓	Sustained weight loss via diet and/or exercise result in reductions in angiogenic factors, and can be maintained up to 30-month follow-up.	Overweight or obesity (n = 439)
Six month running training^[75]^	Plasma Endostatin ↓	Endurance training reduced the antiangiogenic mechanisms by reducing endostatin plasma level	Overweight (n = 21)
12 month moderate-intensityaerobic exercise^[72]^	Plasma PEDF ↓	Fat-loss reduces circulating PEDF in obesity	obesity (n = 173)
LCD VS VLCD diet^[73]^	Circulating VEGF ↑	The rate of weight of loss is positive correlated with angiogenic factor	Obesity (n = 25)
Diet intervention or Exercise intervention^[74]^	Circulating ANT-1 ↓ Circulating and AT ANGPTL4 ↑	weight loss reduced angiogenic activity during obesity	Obesity (n = 79)
Drug intervention			
Exenatide^[79]^	AT angiogenesis ↑ AT1 glyoxalase-1 ↑	Liraglutide improves adipose tissue angiogenic function via GLO	T2DM (n = 140)
Metformin^[92]^	AT TSP-1 ↓	Metformin treatment increases serum TSP-1 in pcos women	PCOS (n = 73)
Rosiglitazone^[86,87]^	AT VEGF-A ANGPTL4 ↑ capillary density ↑	Rosglitazone therapy promotes adipose tissue vascularisation	Obesity (n = 35)
	Serum angiogenin ↑		T2DM (n = 50)

LCD:Low-calorie diet;VLCD:Very low-calorie diet

##### Exenatide

Exenatide, a glucagon-like peptide 1 receptor (GLP-1 R) agonist, is a first-line clinical drug with dual effects of anti-obesity and insulin resistance [77]. Hypoxia of AT drives the occurrence of IR through the disruption of the insulin signalling pathway. The metabolic benefits of the exenatide are correlated with the activation of VEGF-A-mediated angiogenesis and alleviate hypoxia in AT [78]. In line with animal studies, a recent clinical study reported that exenatide increased the vascularization of AT in obese diabetic patients to improve blood glucose levels and insulin sensitivity [79,80]. Therefore, activation GLP-1 R may facilitate AT vascularization, and then improve AT chronic hypoxia to alleviate obesity and obesity-related metabolic disturbance.

##### TNP-470

TNP-470 is a synthetic analogue of fumagillin, which selectively inhibits endothelial cell growth and angiogenesis. The angiostatic mechanism of TNP-470 involves suppression of VEGF production in endothelial cells [65]. Previous studies have focused on that administration of TNP-470 daily reverses obesity by increasing energy expenditure and reducing energy intake [81,82]. However, the mechanism of TNP-470 altering energy balance in AT is not well understood. One possibility is that it is acting directly on the central nervous system and BAT via regulation of blood flow. Future studies are required to confirm the hypothesis. Furthermore, the glucose-lowering effect of TNP-470 in combination with sitagliptin therapy is superior to these drugs alone [83]. However, the clinical potential of TNP-470 remains to be confirmed.

##### Rosiglitazone

PPARγ is a key transcriptional factor for promoting adipocyte differentiation, but enhancing insulin sensitivity. PPARγ induces vascularization to increase adipocyte numbers [84]. Rosiglitazone (RSG), a classic PPARγ agonist, increases capillary density in the AT of obese mice via upregulation of ANGPTL4 and VEGF-A expression [84]. PPARγ-dependent angiogenesis is required for AT healthy growth, whereas PPARγ silencing impairs FA uptake, accelerates ageing and worsen inflammation response by reducing angiogenesis activity in human AdECs [85]. Clinical studies have confirmed that angiogenesis in AT is enhanced temporarily and is accompanied by an increase in adiponectin secretion after RSG administration [86,87]. These evidence suggest that the regulation of glucose and lipid metabolism by PPARγ relies on enhanced vascular density

##### Nifedipine

Nifedipine is a well-recognized dihydropyridine calcium-channel blocker that is widely used for the treatment of hypertension [88]. Experimental studies have revealed that nifedipine has a number of blood pressure-independent effects, including enhanced energy expenditure and resistance to hepatic steatosis [89]. Mechanistically, nifedipine administration ameliorates obesity-impaired vascularization by suppressing oxidative stress and enhancing the number of endothelial progenitor cells [89]. Once the damaged vascularization is repaired by nifedipine, hypoxia of AT will be improved, and mitochondrial respiration of AT will be augmented to prevent the development of obesity. Therefore, nifedipine may be useful in the treatment of obesity-related vascular deficiency.

##### Metformin

Metformin is used as a first-line treatment in newly diagnosed T2DM patients and ameliorates hyperglycaemia and improves systematic metabolism [90]. Although the precise mechanisms of action for metformin in obesity remain unclear, growing studies have approved that metformin also protects cardiovascular system and improves adiposity partly through decreasing angiogenesis activity in AT [91,92]. One prior clinical study believed that metformin-mediated increased angiogenesis activity is related to decreased expression of TSP1 [92], which is a novel antiangiogenic adipokine highly expressed in obese insulin-resistant subjects [93,94]. Altogether, restricting angiogenesis in AT is a potential mechanism for metformin to resist obesity.

##### Botanical extracts

Several plant extracts have been reported to exhibit anti-angiogenic activity by suppression of VEGF-mediated proliferation in ECs [95–97]. For example, ginseng metabolites could suppress adipocyte differentiation and promote apoptosis by decreasing expression of angiogenic factor (VEGF-A, VEGF-B) [95]. Another regulator of angiogenesis and lipogenesis is curcumin polyphenol in turmeric spice, a bioactive extract from Pu-erh tea, which could reduce the adiposity and microvessel density in AT by inhibiting the VEGF signalling pathway [97]. Hence, follow-up studies could commit to developing novel therapeutic and supplementary foods based on the modulation of angiogenesis in AT to combat obesity.

### 4.4 Targeting surface markers of AdECs for obesity treatment

Since prohibitin and ANX2 were identified as surface markers of AdECs useful for its targeting with a peptide precision therapy [98,99], accumulating evidence suggests that the potential of prohibitin or ANX2 as a therapeutic agent against obesity. Mechanistic explanation of ANX2 and prohibitin resisting obesity could be due to accelerated lipids turnover and elevated metabolic rate of adipocytes [100–102]. Herein, targeting surface markers of AdECs translation into potential clinical applications might be feasible.

## 5. Conclusion

Evidence indicating the interaction between adipose-resident cells and AdECs has mined the novel perspective of AT homoeostasis maintenance. We posit that AdECs, as major initiators, effectors, and regulators of metabolic stress, might be a central determinant of unhealthy AT growth. Some metabolites, proteins, miRNAs serve as messengers in the communication between adipocytes and AdECs [103–108]. Recently, one surprising discovery revealed that extracellular vesicles might be carriers of the above-mentioned signal molecules derived from AdECs, which then target adipocytes to adapt to metabolic statues [109]. Hence, the signal transmission between AdECs and adipocytes requires further investigation. Although these fields of exploration remain at an early phase, improving the AdECs health has a high therapeutic potential for obesity. The current challenge is the identification of strategies to specifically target AdECs and modulate their activity, and future studies using microvascular endothelial cells from human AT are warranted.

## Data Availability

The authors confirm that the data supporting the findings of this study are available within the article and its supplementary materials.

## Abbreviations

ADK: denosine kinase; APLNR: atheroprotective peptide apelin receptor; ANGPTL4: angiopoietin-like 4; FGF21: fibroblast growth factor 21; GLP-1R: glucagon-like peptide 1 receptor; HSL: hormone-sensitive lipase; LAL: lysosomal acid lipase; LYVE1: lymphatic vessel endothelial hyaluronan receptor 1; Mdm-2; murine double minute-2; NRG4: neuregulin 4; PDK: 3-phosphoinositide-dependent protein kinase 1; PDGF-CC: platelet-derived growth factor CC; PDK1: 3-phosphoinositide-dependent protein kinase 1; TSP: Thrombospondin; TNP-470: O-(chloracetyl-carbamoyl) fumagillol; VEGF: vascular endothelial growth factor.

## Essential points

- Local endothelial cells in adipose tissues are called AdECs, which communicate with mature adipocytes or adipose stromal cells to maintain adipose tissue homeostasis in a paracrine manner.
- A vast majority of AdECs belong to the type of lymphatic endothelial cells, having a pronounced influence on modulating lipid transport and local inflammation
- Pro- or anti-angiogenic factors from AdECs serve as novel molecular switches for the transformation of white adipocytes to beige adipocytes.
- Pharmacologic angiogenesis intervention is expected to be an effective treatment strategy for obesity.
